# Thrombospondin-1-derived 4N1K peptide expression is negatively associated with malignant aggressiveness and prognosis in urothelial carcinoma of the upper urinary tract

**DOI:** 10.1186/1471-2407-12-372

**Published:** 2012-08-28

**Authors:** Yasuyoshi Miyata, Shin-ichi Watanabe, Hiroshi Kanetake, Hideki Sakai

**Affiliations:** 1 Department of Nephro-Urology, Nagasaki University Graduate School of Biomedical Sciences, Nagasaki, Japan

**Keywords:** Urothelial cancer, Upper urinary tract, 4N1K peptide, Matrixmetalloproteinase-9, Angiogenesis, Prognosis

## Abstract

**Background:**

Thrombospondin (TSP) is a multi-functional protein that appears to have dual roles in cancer, that is, either as a promoter or a suppressor. 4N1K is a TSP-derived peptide that has been reported to be associated with neovascularity, cell survival, and invasion. There is a little information regarding its pathological roles in human cancer tissues. Our aim was to clarify clinical significance and prognostic value of 4N1K expression in patients with urothelial carcinoma of the upper urinary tract (UC-UUT).

**Methods:**

We investigated 4N1K expression in 97 surgically excised, non-metastasized UC-UUT specimens and five normal tissues via immunohistochemistry. Microvessel density (MVD), lymph vessel density (LVD), cancer cell proliferation (PI), apoptotic index (AI), and matrix metalloproteinase (MMP)-9 expression was also determined. The relationships 4N1K expression and pT stage, grade, and prognosis were analysed. In addition, correlations with these cancer-related and TSP-related factors were also investigated.

**Results:**

Strong and moderate 4N1K expression was found in normal urothelial tissues. Of the 97 specimens, 45 patients were positive for 4N1K expression, which was primarily located in the interstitial areas of the cancer tissue. 4N1K expression was negatively associated with pT stage (*p* = 0.003) and grade (*p* = 0.002). Survival analyses revealed that 4N1K is a predictor of metastasis-free (*p* = 0.036) and cause-specific survival (*p* = 0.009). 4N1K expression was closely associated with malignant behaviour, specifically MVD (*p* = 0.001), AI (*p* = 0.013), and MMP-9 expression (*p* = 0.036), but not PI and LVD, as determined via multivariate analysis models.

**Conclusions:**

4N1K expression appears to be associated with cancer cell progression and survival in UC-UUT patients via the regulation of angiogenesis, apoptosis, and MMP-9 expression. There is a possibility that the 4N1K-peptide may be a useful marker and novel therapeutic target in patients with UC-UUT.

## Background

Urothelial cancer (UC) of the upper urinary tract (UUT) is relatively rare, and accounts for only 5% of all UC; however, its prognosis is very poor. For example, the 5-year survival rate of patients with pT4 or metastasis is only 10% [1]. To improve survival and morbidity, numerous reports on the biological and pathological characteristics of this type of cancer have been published [2]. However, more detailed and accurate information regarding its pathological features, biological markers for outcome, and potential therapeutic targets is warranted to improve follow-up and develop treatment strategies for patients with UC-UUT.

Angiogenesis plays a key role in various physiological and pathological processes [3]. Additionally, angiogenesis is important in tumour growth, metastasis, and prognosis in many types of malignancies, including UC [3,4]. Thrombospondin (TSP)-1 is a well-known potent inhibitor of angiogenesis in various physiological and pathological conditions, as it is capable of inhibiting proliferation, migration, and formation of tube-like structures in endothelial cells [5,6]. These anti-angiogenic functions of TSP-1 are also present in various cancers [7]. Conversely, it is thought that TSP-1 can also stimulate angiogenesis in cancer [8]. Indeed, the pro-angiogenic effects of TSP-1 have been previously detected in several cancers including pancreatic cancer and gastric cancer [9,10]. Thus, TSP-1 has opposing angiogenic functions that are dependent on the cancer type.

In recent years, many investigators have paid attention to lymph angiogenesis, as it has been proposed to play important roles in the dissemination of cancer cells and progression of various cancers similar to angiogenesis [11,12]. Several reports demonstrated that TSP-1 is significantly associated with vascular endothelial growth factor (VEGF) function in various cancers [10,13,14]. Furthermore, VEGF was also reported to be associated with lymph angiogenesis in cancer tissues, including UC [3]. Thus, while there is a possibility that TSP-1 is associated with lymph angiogenesis in patients with cancer, there is no information regarding this issue in human cancer tissues.

TSP-1 can also regulate the production of matrix metalloproteinase (MMP)-9 [15,16]. MMP-9 is a type IV collagenase of the MMP family that is capable of cleaving a wide range of extracellular matrix components [17]. In fact, increased MMP-9 levels have been reported to be closely associated with high stages of cancer in various human tissues, including UC-UUT [16,18]. However, as with angiogenesis, TSP-1 appears to have dual roles in the regulation of MMP-9 function in cancer, that is, either as a stimulator [13,15] or an inhibitor [19,20]. In regards to TSP-1 and tumour growth, there are several reports suggesting that TSP-1 may inhibit cancer cell proliferation in several cancers [21,22]. Despite this, TSP-1 was also previously reported to stimulate cell proliferation in colon cancer cells [23]. Thus, TSP-1 is capable of acting as both a stimulator and inhibitor of cancer cell proliferation. It is well-known that TSP-1 can induce apoptosis in endothelial cells [7]. Similarly, several investigators have found that TSP-1 induces apoptosis in malignant cells [24,25]. However, other investigators reported that TSP-1 has anti-apoptotic effects in thyroid cancer [26]. Thus, the direct function and pathological role of TSP-1 in regards to apoptosis of cancer cells is not still fully understood.

There is a wide range of opinions regarding the pathological role, clinical significance, and predictive value of TSP-1 in cancer patients. For example, there are several reports suggesting that low TSP-1 expression is correlated with increases in malignant aggressiveness and poorer outcomes in several cancers, including bladder cancer, prostate cancer, and renal cell carcinoma [14,22,27,28]. Furthermore, high expression of TSP-1 has been associated with high grade, high stage, and poor prognosis in several cancers [13,29,30]. On the other hand, TSP-1 expression has not been associated with the clinicopathological features of renal cell carcinoma and advanced gastric cancer [10,31]. Consequently, the roles of TSP-1 in the clinicopathological features, malignant aggressiveness, and prognosis of various cancers are extremely confusing. Further studies are necessary to reach a consensus on the pathological significance of TSP-1 in various cancers.

TSP-1 is a disulfide-bonded trimer with several different domains. Specifically, it consists of an N-terminal domain, a pro-collagen homology region, CSVTCG sequences within the type I repeats, a RGD sequence within the type 3 repeats, and a C-terminal domain [32]. As mentioned previously, the regulation of TSP-1 function is complex, and involves direct and indirect effects. To clarify the detailed biological and pathological functions of TSP-1, various synthetic peptides derived from TSP-1, such as peptide from the type I repeats (i.e. KRFK and WSHSPW) and peptides from the N-terminal domain (i.e. GQGVLQNVRFVF), have been used [33,34]. Interestingly, one report suggests that the 4N1K peptide (KRFYVVMWKK), which is derived from the C-terminal domain of TSP-1, inhibits angiogenesis both in *in vivo* and *in vitro* models [35]. 4N1K expression was also reported to be negatively associated with angiogenesis in human renal cell carcinoma tissues [31]. However, the clinical and pathological significance of the 4N1K peptide in urothelial cancer (UC) is still unknown.

In the present study, we paid close attention to the pathological role, clinical significance, and prognostic value of 4N1K expression in patients with UC of the upper urinary tract (UC-UUT), as this cancer is characterized by frequent recurrence after initial treatment. Angiogenesis, lymph-angiogenesis, proliferation, apoptosis, and MMP-9 are known to affect the malignant behaviour, tumour progression, and prognosis of UC-UUT [16,18]. Thus, the main goal of the present study was to examine whether 4N1K expression correlates with malignant behaviour, clinicopathological features, and prognosis in patients with non-metastatic UC-UUT.

## Methods

### Patients

Ninety-seven consecutive patients, who were diagnosed with non-metastatic UC-UUT, were reviewed retrospectively. This study included 72 men and 25 women, ranging in age from 39 to 87 years (median age: 67 years). Patients that received any preoperative therapy were excluded. All histological diagnoses, including tumour grade and pT stage, were determined from formalin-fixed and paraffin-embedded specimens obtained from the radical operation. Staging was assessed according to the 2002 tumour-node-metastasis (TNM) classification, and cancer grade was divided into three grades (i.e. G1, G2, and G3), according to World Health Organization (WHO) classification and other recent reports on UC-UUT [36,37]. A single pathologist performed all of the pathological examinations, including lymph and/or blood vessel vascular invasion (LVI), which are assessed by regular hematoxylin and eosin staining. The median follow-up period was 44 months (range: 3–250 months). Fifteen (15.4%) patients experienced metastasis after surgery. In addition, 11 patients had local and/or bladder metastasis after recurrence. Seventy-three (75.3%) patients were alive at the last follow-up examination, while 24 patients (24.7%) had died due to TCC-related disease. The study protocol was approved by the Human Ethics Review Committee of the Nagasaki University Hospital.

### Immunohistochemistry

The methodology for immunohistochemical staining and terminal deoxynucleotidyl transferase-mediated nick and labelling (TUNEL) was previously described elsewhere [11,31]. Briefly, 5-μm-thick sections were deparaffinized in xylene and rehydrated in ethanol. Antigen retrieval was performed for all immunohistochemical staining. All sections were then immersed in hydrogen peroxide to block endogenous peroxidase activity. The primary antibody for 4N1K-containing peptide was previously used by our group, and its specificity was confirmed in several other reports [31,35]. The other antibodies used were obtained from commercial companies. They were as follows: anti-Ki-67 and anti-D2-40 (Dako Corp., Glostrup, Denmark), anti-CD31 (Novocastra, Newcastle, United Kingdom), anti-MMP-9 (Daiichi Fine Chemical, Toyama, Japan), and anti-cleaved caspase-3 (R & D systems, Inc., Abingdon, United Kingdom). Sections were incubated with the primary antibody at 4 °C overnight. After incubation with the primary antibody, the sections were washed extensively, and then treated with peroxidase using the labelled polymer method with DAKO EnVision + ^TM^ Peroxidase (Dako Corp., Carpinteria, CA). The peroxidase reaction was visualized with the liquid DAB substrate kit (Zymed Laboratories Inc., San Francisco, CA). Sections were counterstained with hematoxylin, dehydrated stepwise through a graded alcohol series, and cleared in xylene before mounting. A consecutive section from each sample processed without the primary antibody was used as a negative control. Positive controls were similar to those used in previous reports. To evaluate the apoptotic cells, we determined two parameters, the proportions of cleaved caspase-3- and TUNEL-positive cells. The method of *in situ* labelling for apoptosis was performed as previously described [38]. We used the Apop Tag *In Situ* Apoptosis Detection Kit (Intergen Company, Purchase, NY), which is based on TUNEL.

Immunohistochemical staining was assessed with light microscopy, where staining intensity was graded as none, weak, moderate, or strong. Carcinoma cells that demonstrated moderate or strong staining were considered to be positively stained cells. The evaluations of immunohistochemical staining for Ki-67, CD31, and MMP-9 were performed as previously described [18,38]. For all variables, values above the median were considered as the higher group, and those with staining equal to or less than the median value were considered as the lower group for statistical analyses, including logistic regression analyses. Immunoreactive staining was evaluated using the semi-quantification method. To ensure accuracy and validity, slides were blindly evaluated twice at different times by two investigators (Y.M. and S.W.). All specimens were examined using a Nikon E-400 microscope and digital images were captured (Nikon DU100, Tokyo, Japan). Additionally, we used a computer-aided image analysis system (Win ROOF, version 5.0, MITANI, Fukui, Japan) to calculate the statistical variables.

### Statistical analyses

Normality was evaluated via a normal distribution and histograms for each variable. Data are expressed as medians (interquartile range), unless otherwise stated. The Mann–Whitney *U* test was performed for continuous variables, and the chi-square test was used for categorical comparison of the data. The crude and adjusted effects on tumour stage and grade, as well as other risk factors, were estimated by logistic regression analysis, and were described as odds ratios (OR) with 95% confidence intervals (95% CI), together with the *p* values. Variables that achieved statistical significance in the univariate analysis were subsequently included into a multivariate analysis model. The metastasis-free and cause-specific survival rates were compared with Kaplan-Meier analysis and a log rank test. Variables that achieved statistical significance (*p* < 0.050) in the univariate analysis were subsequently entered into a multivariate analysis using a COX proportional hazards analysis, and results were described as hazard ratio (HR) with 95% CI and the *P* values. All statistical tests were two-sided and significance was defined as *p* < 0.050. All statistical analyses were performed on a personal computer with the statistical package, StatView for Windows (Version 5.0, Abacus Concept, Inc., CA).

## Results

### 4N1K expression

**Figure 1:**
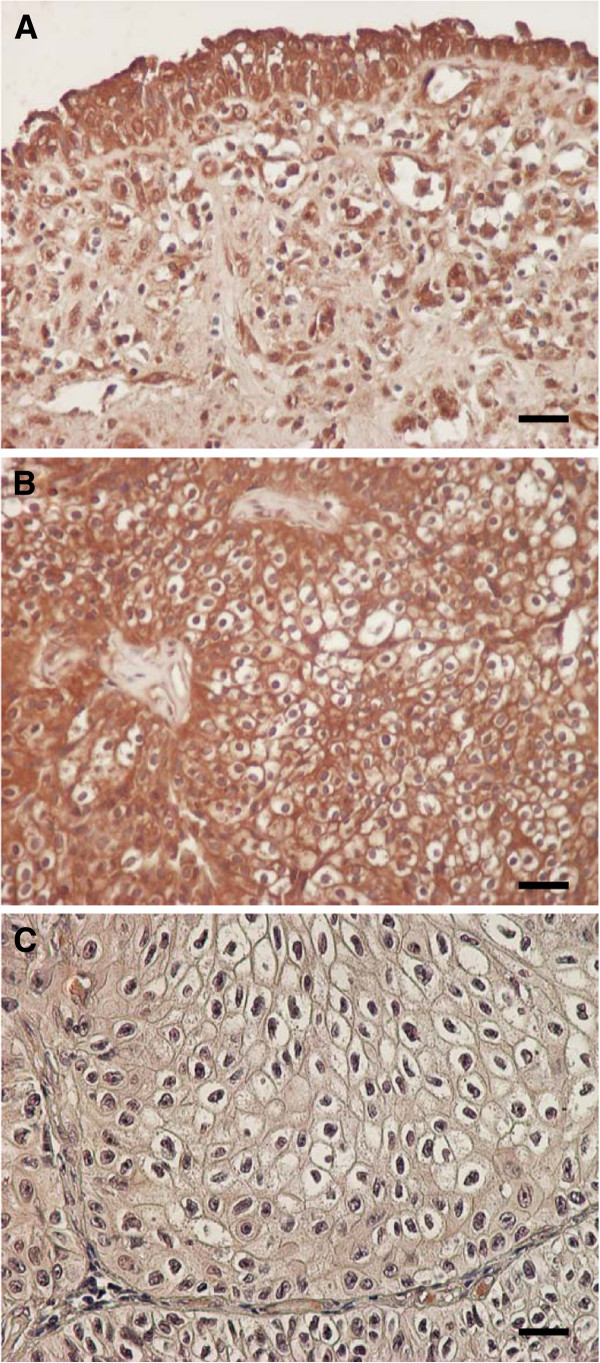
**(A) In normal urothelial cells, strong or moderate 4N1K expression was detected in all specimens.** (**B**) In cancer tissues, some specimens demonstrated strong or moderate 4N1K expression. This figure was a representative example of strongly stained cancer cells (**C**) A representative image of weakly stained tissues for 4N1K peptide. All sections were counterstained in hematoxylin (Magnification: ×400; Bar means 10 μm)

Representative examples of 4N1K expression are presented in Figure 1. Immunostaining for this peptide was primarily detected in the intestinal tissues, and, in part, within the cytoplasm of cells. In addition, some stromal cells, including infiltrating, endothelial, and fibroblast-like cells, showed 4N1K immunostaining. Strong and moderate expression of the 4N1K peptide was found in normal urothelial tissues of all 5 specimens (Figure 1A). Representative examples of strong expression judged to be high, and weak expression judged to be low, are presented in Figures 1B and C, respectively. Forty-five patients (47.4%) were judged to have high expression of the 4N1K peptide. We noticed that this peptide demonstrated a trend of being strongly expressed near neighbouring stromal tissues, including blood vessels in cancer tissues. However, clear characteristics regarding the distribution of 4N1K expression were not evident.

### Correlation with clinicopathological features

**Figure 2:**
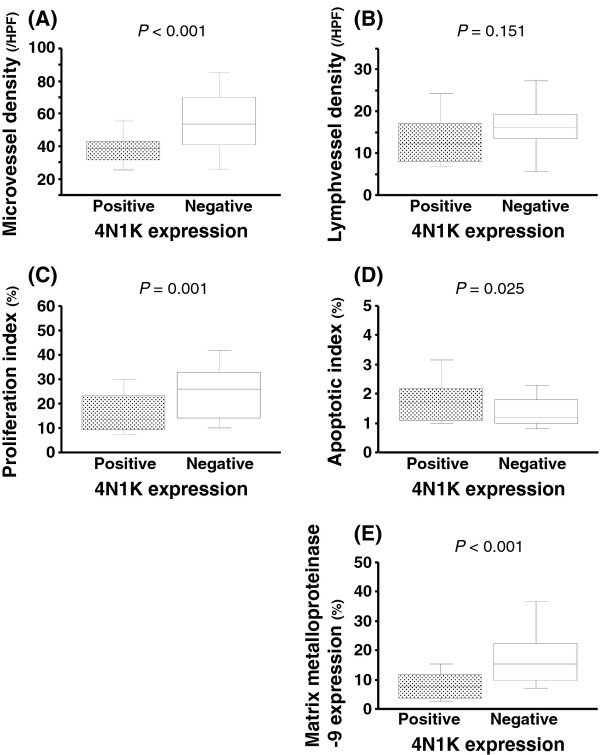
Correlations between 4N1K expression and (A) microvessel density (MVD), (B) lymph-vessel density (LVD), (C) cancer cell proliferation index (PI), (D) apoptotic index (AI), and (E) matrix metalloproteinase-9 expression (MMP-9)

The correlations between 4N1K expression and pathological features are presented in Table 1. In non-muscle invasive cancer (NMIBC; pTa + 1), 28 of 43 patients (65.1%) were judged as positive. Conversely, in muscle invasive disease, its ratio (18 of 54 patients = 33.3%) was significantly lower (*p* = 0.002) compared to NMIBC. With respect to grade, there was a similar trend, where 4N1K expression negatively correlated with the grade (*p* = 0.001). To further determine the function of 4N1K expression, univariate and multivariate analyses were performed. Using univariate analyses, it was found that, with the exception of LVD, all of the other factors were associated with 4N1K expression (Figure 2A-E). Furthermore, it was found that 4N1K expression independently correlated with MVD (OR = 4.01; 95% CI = 1.48-10.88; *p* = 0.001), AI (OR = 2.39; 95% CI = 1.06 – 5.42; *p* = 0.036), and MMP-9 expression (OR = 3.36; 95% CI = 1.30-8.69; *p* = 0.013), as determined via multivariate analysis models, which included pT stage and grade.

**Table 1:** Pathological features and 4N1K expression

**parameters**	**n**	**4N1K expression**		***p*** **value**
		**Negative (%)**	**Positive (%)**	
pT stage				
Ta	7	2 (28.6)	5 (71.4)	0.003
T1	36	13 (36.1)	23 (63.9)	
T2	17	7 (41.2)	10 (58.8)	
T3	27	21 (77.8)	6 (22.2)	
T4	10	8 (80.0)	2 (20.0)	
Low (Ta + 1)	43	15 (34.9)	28 (65.1)	0.002
High (T2-4)	54	36 (66.7)	18 (33.3)	
Grade				
Low grade	55	21 (38.2)	33 (61.8)	0.001
High grade	42	30 (71.4)	12 (28.6)	

### Correlation with survival

**Figure 3:**
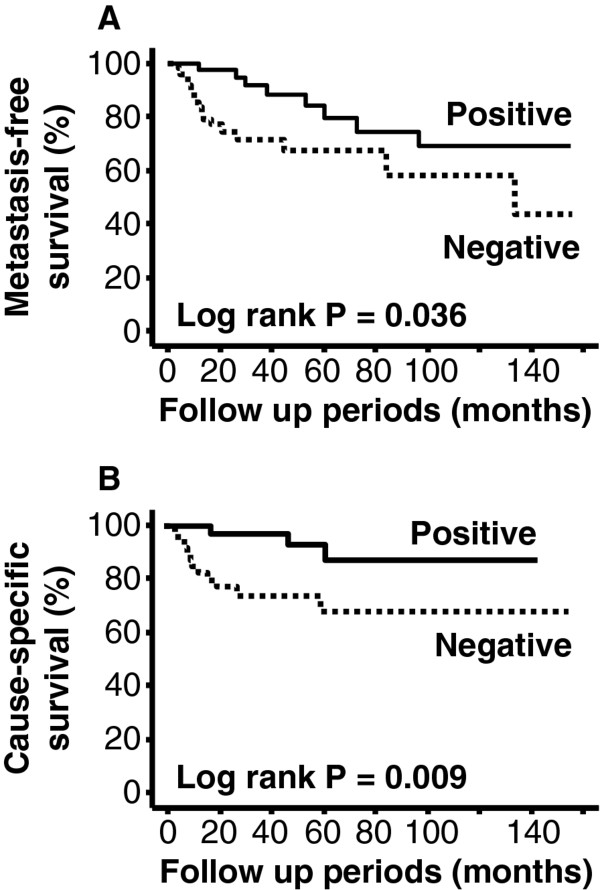
**Kaplan-Meier survival curves according to 4N1K expression status.** Patients with negative expression had poorer (**A**) metastasis-free (*p* = 0.036) and (**B**) cause-specific (*p* = 0.009) survival compared to those with positive expression

No significant relationship was observed between 4N1K expression and site of metastasis. Survival analyses demonstrated that negative expression of 4N1K peptide is a poor prognostic factor for metastasis-free survival (*p* = 0.036, Figure 3A) and cause-specific survival (*p =* 0.009, Figure 3B) in our study population. Similar analyses also revealed that metastasis-free survival and cause-specific survival were associated with pT stage (*p* < 0.001 and *p* = 0.001) and grade (*p* = 0.001 and *p* = 0.006). Initially, due to our limited sample size, we used a simple multivariate analysis model, which included these pathological features. As shown in Table 2, these analyses revealed that 4N1K expression was not an independent predictor of metastasis-free survival and cause-specific survival. On the other hand, in both analyses, only high pT stage was recognized as an independent predictor of metastasis-free and cause-specific survival (Table 2). Additionally, we performed further multivariate analyses which included the two former pathological parameters and adjuvant therapy, LVI, PI, MVD, and MMP-9 expression since all of these factors were significantly associated with 4N1K expression and survival in univariate analyses. These analyses showed that only high pT stage and presence of LVI were independent predictors (HR =9.16; 95%CI = 1.02 – 82.58; *p* = 0.048 and HR = 9.41, 95%CI = 1.91 – 46.42; *p* = 0.006) of metastasis-free survival. However, none of these factors were independent predictors of cause-specific survival in the multivariate analysis model.

**Table 2:** Multivariate analysis of metastasis-free and cause-specific survival

	**For metastasis-free survival**			**For cause-specific survival**		
	**HR**	**95% CIs**	***p*value**	**HR**	**95% CI**	***p*value**
pT stage	8.96	1.08 – 74.43	0.042	3.14	1.08 – 9.19	0.036
Grade	2.12	0.62 – 7.24	0.230	1.75	0.68 – 4.50	0.036
4N1K expression	0.40	0.11 – 1.54	0.186	0.70	0.27 – 1.81	0.463

HR; hazard ratio, CIs; confidential intervals.

## Discussion

Our findings demonstrate that 4N1K peptide expression in tumour tissues of UC-UUT is negatively associated with malignant potential and tumour progression. Additionally, lower expression levels of this peptide were also associated with metastasis and survival in patients with UC-UUT. These observations corroborate those of a previous report on patients with renal cell carcinoma [31]. TSP-1 function is affected by the cellular phenotype via interactions with various cell surface proteins, and the status of its microenvironment [7]. For example, biological activities on cell invasion and focal adhesion of TSP-1 were depended on its concentration [39]. Moreover, matrix-binding TSP-1 was shown to be influenced by angiogenic function *in vitro* compared to non-binding TSP-1 [40]. Thus, TSP-1 appears to have a wide range of the biological functions and pathological roles, and consequently, TSP-1 protein expression appears to be an inadequate and unstable marker for the prediction of pathological features and prognosis in patients with UC-UUT. Indeed, the pathological role and prognostic value of TSP-1 expression in patients with UC of the urinary bladder is controversial. For example, several reports demonstrated that TSP-1 expression is associated with its clinicopathological features, including grade, pT stage, and lymph node metastasis, as well as prognosis [14,16]. Conversely, other studies found that TSP-1 expression is not associated with both the grade and stage of cancer, but rather with disease progression, including disease recurrence and overall survival [27,41]. Another report found that TSP-1 expression is associated with tumour grade, stage, and size, but not with disease progression [42]. This study also showed that the pathological significance of TSP-1 was different in cancer cells and stromal tissues.

Synthetic peptides derived from TSP-1 have been used in previous studies for the investigation of its physiological functions and pathological roles. In the present study, we focused on the expression of the 4N1K peptide for the following reasons: i) antibodies against this peptide were shown to block the TSP-1-induced inhibition of angiogenesis, suggesting that 4N1K plays a crucial role in the inhibitory effects of TSP-1 on angiogenesis [35]; and ii) 4N1K expression was found to be closely associated with malignant potential, cancer cell progression, and survival in patients with renal cell carcinoma [31].

Currently, there is no general agreement on the distribution and production of TSP-1 in UC. While some investigators found TSP-1 expression in tumour stromal tissues, its expression was weak or absent in bladder cancer cells [43]. Conversely, other investigators demonstrated TSP-1 expression in both bladder cancer cells and tumour stromal tissues [27,42]. Additionally, one report found that TSP-1 expression was detected only within the cytoplasms of bladder cancer cells [16]. In bladder cancer cell lines, TSP-1 expression was reported to be expressed in MGH-U4 and RT-4 cell lines, but not in RT-112 and UMUC-3 cell lines [44]. Thus, TSP-1 in UC is thought to be secreted from cancer and/or stromal cells. Based on these observations, we speculated that some factors, including a variety of proteases within interstitial tissues, may break down the 4N1K peptide in human UC-UUT tissues.

One of the most interesting findings of the present study is that 4N1K expression is closely associated with MVD, cancer cell apoptosis, and MMP-9 expression, as determined via multivariate analyses. With respect to the relationship between TSP-1 and angiogenesis, several studies have previously demonstrated that its expression was significantly associated with MVD in human cancer tissues, including bladder cancer [10,22,41]. Moreover, there are a few reports on the relationship between 4N1K peptide and MVD in human cancer tissues. One report found that the 4N1K protein is capable of inhibiting tube formation *in vitro*, as well as angiogenesis in mouse cornea [35]. Additionally, in renal cell carcinoma, 4N1K expression was found to be associated with angiogenesis. These observations corroborate our finding that the 4N1K peptide is closely associated with the regulation of angiogenesis in patients with UC-UUT. Despite this, we also found that 4N1K expression was not associated with lymph angiogenesis. This is the first ever study to report on the relationship between 4N1K expression and LVD in human cancer tissues. Interestingly, similar findings were demonstrated with TSP-1. Specifically, it was found that increases in the expression of TSP-1 inhibited angiogenesis, but did not affect lymph angiogenesis in mice [45].

There is little information available regarding the relationship between 4N1K peptide expression and cancer cell apoptosis in human cancer tissues. It has been suggested that CD47/integrin-associated protein (IAP)-binding protein play an important role in the apoptosis of various cells [7,46]. The 4N1K peptide has also been shown to induce apoptosis-like cell death in immune cells [47]. Furthermore, 4N1K expression was previously shown to be positively associated with AI in human renal cell carcinoma tissues [31]. These findings support our observations, specifically that the 4N1K peptide may have pro-apoptotic functions in patients with UC-UUT. However, another peptide, 4 N1 (RFYVVMWK), which is also derived from the CD47/IAP-binding domain and has a similar structure to the 4N1K peptide (KRFYVVMWKK), has been shown to inhibit apoptosis of human thyroid cancer cells [26]. Thus, further studies are necessary, as the biological activities of these peptides appear to be dependent on the cell type and situation.

Another interesting finding of the present study was that a negative expression of 4N1K peptide was significantly and closely associated with an elevated expression of MMP-9, as determined via multivariate analysis. The regulation of MMP-9 expression and function through TSP-1 is very complex. For example, a low dose of TSP-1 stimulates its expression, whereas a high dose TSP-1 inhibits its expression [48]. Additionally, certain peptides derived from TSP-1, such as type I repeats [19] and CSVTCG sequence [13], are known to bind to MMP-9 and inhibit its activation. In regards to the role of TSP-1 in regulating MMP-9 in cancer cells, there were several opinions that TSP-1 acts as an inhibitor in breast cancer [19] and ovarian cancer [20]. In UC, TSP-1 expression was found to be negatively correlated with MMP-9 expression [16]. Although there are no reports on the effects of 4N1K on MMP-9 expression in human cancer patients, these findings support our observation. However, there is one report that found that the 4N1K peptide up-regulates MMP-9 in neurovascular cells [49]. Thus, more detailed studies are necessary to determine the pathological function of the 4N1K peptide in the regulation of MMP-9 expression in UC.

Our study has the limited sample size and variables were evaluated by semi-quantification. So, further investigations are necessary to clarify the biological activities and pathological function of 4N1K peptide in UC-UUT. However, our results were important information to discuss the clinical significance of 4N1K peptide expression in these patients.

## Conclusions

Our results showed that 4N1K expression was negatively associated with malignant potential and tumour growth in patients with UC-UUT. In addition, patients with a negative expression of 4N1K had a poorer prognosis for metastasis and cause-specific survival. We speculated that 4N1K expression may affect to these malignant behaviour and prognosis via regulation of angiogenesis, apoptosis, and MMP-9 expression. Additionally, our observations are also useful for discussing follow-up and treatment strategies in patients with UC-UUT.

## Competing interests

The authors declare no competing interests.

## Authors’ contributions

SW and YM designed the histochemical experiments, performed experiments, and drafted the manuscript. HK and HS participated in the design of the study. YM participated in the overall design, study coordination and finalized the draft of the manuscript. All authors read and approved the final manuscript.

## Pre-publication history

The pre-publication history for this paper can be accessed here:

http://www.biomedcentral.com/1471-2407/12/372/prepub
